# Characteristics of soil microbiota and organic carbon distribution in jackfruit plantation under different fertilization regimes

**DOI:** 10.3389/fmicb.2022.980169

**Published:** 2022-09-20

**Authors:** Lanxi Su, Tingyu Bai, Gang Wu, Qingyun Zhao, Lehe Tan, Yadong Xu

**Affiliations:** ^1^Spice and Beverage Research Institute, Chinese Academy of Tropical Agricultural Sciences, Wanning, Hainan, China; ^2^National Tropical Plants Germplasm Resource Center-Sub Centre of Germplasm Resource for Woody Grain, Wanning, Hainan, China; ^3^Key Laboratory of Genetic Improvement and Quality Regulation for Tropical Spice and Beverage Crops of Hainan Province, Wanning, Hainan, China; ^4^School of Agricultural Sciences, Zhengzhou University, Zhengzhou, China

**Keywords:** fertilization, soil organic carbon component, soil quality, microbial and nematode community, function prediction

## Abstract

Manure amendment to improve soil organic carbon (SOC) content is an important strategy to sustain ecosystem health and crop production. Here, we utilize an 8-year field experiment to evaluate the impacts of organic and chemical fertilizers on SOC and its labile fractions as well as soil microbial and nematode communities in different soil depths of jackfruit (*Artocarpus heterophyllus* Lam.). Three treatments were designed in this study, including control with no amendment (CK), organic manure (OM), and chemical fertilizer (CF). Results showed that OM significantly increased the abundance of total nematodes, bacterivores, bacteria, and fungi as well as the value of nematode channel ratio (NCR) and maturity index (MI), but decreased plant-parasites and Shannon diversity (H′). Soil microbial and nematode communities in three soil depths were significantly altered by fertilizer application. Acidobacteria and Chloroflexi dominated the bacterial communities of OM soil, while Nitrospira was more prevalent in CF treatment. Organic manure application stimulated some functional groups of the bacterial community related to the C cycle and saprotroph-symbiotroph fungi, while some groups related to the nitrogen cycle, pathotroph-saprotroph-symbiotroph and pathotroph-saprotroph fungi were predominated in CF treatment. Furthermore, OM enhanced the soil pH, contents of total soil N, P, K, and SOC components, as well as jackfruit yield. Chemical fertilizers significantly affected available N, P, and K contents. The results of network analyses show that more significant co-occurrence relationships between SOC components and nematode feeding groups were found in CK and CF treatments. In contrast, SOC components were more related to microbial communities than to nematode in OM soils. Partial least-squares-path modeling (PLS-PM) revealed that fertilization had significant effects on jackfruit yield, which was composed of positive direct (73.6%) and indirect effects (fertilization → fungal community → yield). It was found that the long-term manure application strategy improves soil quality by increasing SOM, pH, and nutrient contents, and the increased microbivorous nematodes abundance enhanced the grazing pressure on microorganisms and concurrently promoted microbial-derived SOC turnover.

## Introduction

Soil is the most active carbon pool in the ecosystem, with an organic carbon stock of 1,500 Gt in the first meter (Balesdent et al., 2018). Soil organic carbon (SOC) is an important indicator for soil quality assessment because it contributes to the modification of biological, physical, and chemical properties of soil (He et al., 2015). Due to the large variations in environmental conditions (geography and climate) as well as the background of relatively stable soil organic C, it is difficult to detect the changes of SOC in the short and medial term (Haynes, 2005). Physicochemical properties and turnover time influence the degree of stabilization of SOC components. Labile organic carbon (LOC) is a small component of SOC, which includes potassium permanganate-oxidizable C (KMnO_4_-C), microbial biomass carbon (MBC), and dissolved organic carbon (DOC) (Haynes, 2005). The labile C fraction responds to fertilization management more quickly than SOC, and exhibits rapid turnover times (Mi et al., 2019). Hence, these components are considered early indicators of soil quality and affect soil function in specific ways (Blanco-Moure et al., 2016). Measurement of a single fraction of LOC does not adequately reflect the changes in soil quality caused by management. Instead, it is necessary to measure several LOC components simultaneously to estimate the effects of management on soil properties.

The application of organic manure (OM) (e.g., residues, compost, and manure) is an effective way to increase soil C storage through direct C inputs and/or indirect increase in net primary productivity and root litter and exudation, which contributes mostly to soil sequestered or stable C and the composition of the soil microbiota (Sokol and Bradford, 2019; Lazcano et al., 2021). Different feeding habits of nematodes affect the composition and function of soil microbial communities (Liu et al., 2018). Generally, the top-down regulation of predators by microfauna positively influences the microbial biomass and community structure (Neher, 2010). The highly complex network between nematodes and microbes in soil plays a vital role in SOC conversion (Jiang et al., 2018). The composition and abundance of microbes that release plant-available nutrients from organic fertilizers was strongly correlated with the mineralization of organic carbon (Zhang H. et al., 2015). However, it is still difficult to explain the relationship between microbiota and SOC components when soils are amended with exogenous organic resources.

The composition of SOC in surface soils and its association with environmental variation has been extensively investigated over the years (Doetterl et al., 2015). In a 26-year application of fertilization strategies, Li et al. (2018) reported that OM can increase the concentrations and proportions of labile C as well as the stock of stable C in topsoil. Qaswar et al. (2020) reported that the 34-year application of manure and inorganic fertilizers increased crop yield sustainability and the organic carbon sequestration rate in the top layer. By comparison, soils deeper than 20 cm below ground contain more than half of global SOC pools (Rumpel et al., 2012). Furthermore, microbial community structure, carbon availability, and composition often change with soil depth (Stone et al., 2014). Nevertheless, the composition and preservation of SOC components in deeper layers are poorly understood, especially when it comes to the stability and function of soil biota in tropical agroecosystems.

Over the last 20 years, jackfruit (*Artocarpus heterophyllus* Lam.) has been widely cultivated in tropical and subtropical regions of China due to its high economic benefits. The widespread and inappropriate fertilization regimes [e.g., excessive chemical fertilizer (CF) inputs] have adverse effects on soil C sequestration due to the acceleration of C mineralization (Brown et al., 2014). Organic amendments and the replacement of CF are increasingly recommended as effective measures to supplement soil C sources in orchards (Maltas et al., 2018). As a deep-rooted fruit, its main absorption roots are distributed in the 0–60 cm soil layer. In this study, we focused on the effects of long-term OM on soil microbial and nematode communities as well as organic carbon distribution in different soil layers. We aimed (1) to investigate the distribution of total and labile organic C in the three layers of soil depths (0–20, 20–40, and 40–60 cm) under different fertilization patterns; (2) to evaluate the impact of different fertilization patterns on the abundance and composition of the soil microbiota; (3) to explore and describe the relationships among various components of SOC, soil microbial and nematode communities.

## Materials and methods

### Experimental design and sample collection

The long-term fertilization experiment commenced in the town of Gaolong in Wanning City, Hainan Province, China (18°737′N, 110°192′E) with an 8-year jackfruit monoculture, including three treatments with triplicates in a random plot design. The individual plot of each treatment consists of 20 jackfruit trees with 450 m^2^ (25 m × 18 m). Three treatments, OM, CF, and control (CK, without any amendment), were applied to the field since jackfruit was planted. Chemical fertilizer treatment was adjusted to the same amounts of N, P, and K as OM by applying urea, calcium magnesium phosphate, and potassium chloride, respectively (Table 1). Information about the study site and the characteristics of manure used had been described in detail in our previous manuscript (Su et al., 2021).

**TABLE 1:** The amounts of the applied fertilizers for each treatment per year.

Treatment	Fertilizer amount (kg/plant)			
	Organic manure	Urea	Calcium magnesium phosphate	Potassium chloride
CK	0	0	0	0
CF	0	5	39	3
OM	150	0	0	0

CK, no fertilization; CF, chemical fertilization; OM, organic manure.

To evaluate the reproducibility of the experiment, a total of fifty-four soil samples (3 treatments × 3 depths × 3 biological replicates × 2 sampling times) in three different layers of depths (0–20, 20–40, and 40–60 cm) were collected from six random sites under the trunk base of each tree of the treatment plots on June 2019 and 2020. Composite samples of six sites per plot were collected with a shovel. Each sample was collected in an independent sterile plastic bag, sealed, and homogenized thoroughly. Taxonomic analysis of nematodes was classified from about 200 g of fresh soil samples, chemical analyses were chosen from 100 g of soil samples after air-dried and sieved (<1 mm), and soil DNA was extracted from 50 g of soil samples after gently sieved through a 2 mm sieve and stored at −80°C. The total jackfruit fruit yield in each treatment was weighed from all harvested mature jackfruit fruits in each plot.

### Soil nematode determination

A modified cotton-wool filter method was used for nematode extraction. The number of nematodes was expressed as the number of individuals per 100 g dry soil. And at least 100 nematodes were randomly selected from each sample and identified as four trophic groups: bacterivores (Ba), plant-parasites (Pp), fungivores (Fu, and omnivores-predators (Op) (Yeates et al., 1993). In the case where the number of total nematodes did not reach 100 in a sample, all nematodes were identified. The guilds were characterized on the colonizer-persister (c-p) scale (1–5) as previously described (Bongers and Bongers, 1998). The ecological indices of soil nematodes had been described in our previous manuscript (Su et al., 2021) and calculated as follows: maturity index (MI) and Shannon diversity (H′) for genera, and nematode channel ratio (NCR) for detecting the decomposition pathways of soil organic matter. We visualized the potential differences of nematode communities in soil using principal coordinate analysis (PCoA) based on the Bray–Curtis different similarity matrix generated on nematode abundance. The effect of fertilization on the nematode community structure was studied using a permutational multivariate analysis of variance (PERMANOVA) with 999 permutations by the Adonis function (vegan package) in R (Ginestet, 2011).

### DNA extraction, quantification of the total soil microbial biomass and Illumina sequencing

PowerSoil™ DNA Isolation Kit (MoBio Laboratories Inc., Carlsbad, CA, United States) was used to extract total DNA from 0.25 g of soil, as directed by the manufacturer’s instructions. The quality of DNA was detected with a spectrophotometer (NanoDrop 2000, United States). The total numbers of soil bacteria and fungi were quantified by Real-Time PCR and performed according to the methods described by Su et al. (2021). The bacterial 16S rRNA gene V4 hypervariable region was amplified with primers 520F and 802R (Claesson et al., 2009) from soil genomic DNA, while fungal ITS1 was amplified using primers ITS1F and ITS2R (Mueller et al., 2014). The sequencing was performed using the Illumina MiSeq PE250 sequencing platform (Illumina, Inc., CA, United States) at Personal Biotechnology Co., Ltd., Shanghai, China. The sequence data were made available in the National Center for Biotechnology Information (NCBI) Sequence Read Archive (SRA) database under BioProject number PRJNA836735.

### Bioinformatics analyses

The adaptors and primer sequences were removed, and the raw sequences were demultiplexed according to a unique barcode. Paired-end reads for all samples were run through Trimmomatic (version 0.35) to remove low-quality base pairs according to the parameters (SLIDINGWINDOW: 50:20 MINLEN: 50). The trimmed reads were merged using FLASH program (version 1.2.11) with default parameters (Magoč and Salzberg, 2011). Briefly, low-quality sequences were removed according to screen.seqs command using the following filtering parameters, maxambig = 0, minlength = 100, maxlength = 580, maxhomop = 8. The reserved sequences were assigned to operational taxonomic units (OTUs) with a threshold of 97% identity level using the UPARSE pipeline (Edgar, 2013). Taxonomic assignment was performed using SILVA reference database (v12_8) (Quast et al., 2013) and UNITE database (v7.0) (Kõljalg et al., 2013) for bacteria and fungi, respectively, with a confidence score ≥0.6 by the classify.seqs command in mothur (Schloss et al., 2009). The taxonomic information of operational taxonomic unit (OTU) (from Phylum to Species) was classified based on NCBI. The alpha-diversity was estimated using the Chao1 richness, Shannon, and phylogenetic diversity indices which were calculated based upon neighbor-joining phylogenetic trees generated with mothur and plotted by R. To explore major similarity and variance components of soil microbial community structures, PCoA based on Bray–Curtis distance was performed on OTUs matrices and sample grouping data in R. Permutational multivariate analysis of variance was performed to evaluate the effect of fertilization and soil depths on microbial community structure (Kusstatscher et al., 2020). To visualize the associations among nematodes, bacteria, fungi, and SOC components in the network interface, a correlation matrix was used to calculate the possible pairwise Spearman’s rank correlations. The distribution matrix of total nematodes genera, bacteria, and fungi phyla into the relative abundance for network construction was standardized. A valid co-occurrence was considered a statistically robust correlation between taxa with the Spearman’s correlation coefficient (rho) >0.6 and the *P*-value <0.01 (Shannon et al., 2003). The network analyses and topological characteristics of the networks were performed using R and calculated as the methods described by Su et al. (2021). A partial least squares path model (PLS-PM) was carried out with SmartPLS (Sarstedt and Cheah, 2019) to evaluate the direct and indirect effects of fertilization, SOC components (SOC, POC, DOC, and MBC), microbial and nematode community composition on the jackfruit yield. Microbial and nematode compositions were used as a latent variable, reflecting the relative abundance of each phylum and trophic group, respectively. The goodness of fit of the PLS-PM was evaluated by examining the Goodness-of-Fit index and the coefficient of determination (*R*^2^) of the latent variables. For investigating the functions of the bacterial and fungal communities, Functional Annotation of Prokaryotic Taxa (FAPROTAX) and FUNGuild were used for the identification of potential functions in different treatments *via* the default settings based on taxonomic information of microorganisms, respectively (Louca et al., 2016).

### Determination of soil physicochemical properties

A glass electrode meter was used to determine the soil pH at a ratio of 1:5 after 30 min of shaking. Soil organic carbon was measured with the potassium dichromate external heating method. Dissolved organic carbon was measured with Micro 2,000 N/C Analytic Jena (Jones and Willett, 2006). Potassium permanganate-oxidizable carbon (POC) was measured as described by Blair et al. (1995). Microbial biomass carbon was analyzed using the fumigation-extraction method (Vance et al., 1987). Total soil nitrogen (TN) and alkalyzable nitrogen (AN) contents were measured with Kjeldahl digestion and alkaline-hydrolyzable diffusion method, respectively. Total phosphorus concentration (TP) was determined by digesting soil samples in acid (HClO_4_–H_2_SO_4_), followed by estimation on a spectrophotometer after developing a yellow color using the molybdenum blue method. The concentration of available phosphorus (AP) was extracted with sodium bicarbonate and then measured with the method of molybdenum blue. Total potassium concentration (TK) was extracted by the sodium hydroxide melting method and determined by a flame photometer. The concentration of readily available potassium (AK) was determined by flame photometry after extraction with ammonium acetate.

### Statistical analyses

SPSS version 20.0 statistical software (SPSS Inc., Chicago, IL, United States) was used to perform a one-way analysis of variance (ANOVA), and Duncan multiple range tests on all parameters in the site to examine difference significance at a value of *P* < 0.05. The figures were prepared in Origin 2016 and the results were reported as mean ± standard error (SE).

## Results

### Fertilization affects nematode community assembly in different soil depths of jackfruit

The prevalent taxa number and abundance of nematode confirmed the impact of OM on the nematode community (Supplementary Tables 1, 2). The genus of *Cephalobus* and *Mesorhabditis* that belongs to bacterivores, and the *Tylencholaimus* that belongs to fungivores were more distributed in OM treatment in both two field experiments. Moreover, a significantly higher number of bacterivores (*Geomonhystera* and *Acrobeloides*) and omnivores-predators (*Labronema*) was observed in OM soil (*P* < 0.05), while a lower number of plant-parasites (*Rotylenchulus*, *Meloidogyne*, and *Tylenchorhynchus*) was detected in the second-year experiment. There was no obvious distinction in variation among different soil depths under the same treatment.

Organic manure significantly increased total nematodes in different soil depths compared with other treatments (Table 2). For the trophic groups, bacterivores and fungivores were significantly enriched in OM treatment in soil depths of 0–20 and 20–40 cm in the first-year experiment, respectively (*P* < 0.05). Bacterivores, fungivores and omnivores-predators were relatively more abundant in OM soil, and the number of plant-parasites in all soil layers was the lowest in the second year experiment.

**TABLE 2:** Effect of organic manure (OM) and chemical fertilization treatments on the relative abundance of trophic groups (%) of nematode and ecology indices in the field experiment.

Sampling time	Soil depth (cm)	Treatment	Bacterivores	Fungivores	Plant-parasites	Omnivores-predators	Total nematodes	MI	H′	NCR
2019	0–20	CK	14.5b (4.62)	28.7a (2.76)	33.6a (4.47)	23.2a (5.10)	89b (9.21)	2.35a (0.12)	2.33a (0.08)	0.33a (0.08)
		CF	12.1b (2.35)	16.3a (4.98)	48.3a (5.94)	23.2a (3.61)	89b (8.82)	1.75a (0.25)	2.13a (0.04)	0.45a (0.12)
		OM	35.2a (7.67)	22.2a (3.66)	27.3a (9.43)	15.3a (1.93)	145a (6.24)	2.12a (0.25)	2.27a (0.05)	0.6a (0.08)
	20–40	CK	26.9a (14.38)	11.1b (2.26)	34.4a (6.18)	27.6a (8.22)	95b (9.06)	1.99a (0.16)	2.12a (0.22)	0.64a (0.12)
		CF	20.5a (4.05)	9.2b (2.37)	52.0a (10.85)	18.3a (4.74)	32c (2.19)	1.46a (0.38)	2.27a (0.09)	0.69a (0.03)
		OM	35.3a (4.55)	20.3a (1.90)	36.6a (3.52)	7.8a (1.10)	141a (16.90)	1.73a (0.03)	2.05a (0.14)	0.63a (0.05)
	40–60	CK	15.6a (4.48)	26.7a (5.55)	34.9a (14.63)	22.8a (5.44)	23c (2.85)	2.19a (0.45)	1.96a (0.10)	0.36b (0.04)
		CF	23.9a (1.03)	13.3a (3.65)	48.9a (1.96)	13.9a (5.63)	82b (9.84)	1.49a (0.14)	1.98a (0.07)	0.65a (0.06)
		OM	21.3a (4.64)	25.0a (3.56)	38.8a (2.53)	14.9a (2.62)	132a (13.32)	2.04a (0.20)	2.06a (0.06)	0.46ab (0.09)
2020	0–20	CK	9.2b (2.15)	6.7b (1.34)	58.8a (4.32)	25.4b (2.03)	91c (5.17)	1.52b (0.13)	2.14a (0.17)	0.57b (0.03)
		CF	15.3b (2.59)	5.4b (1.26)	61.4a (6.60)	17.9b (5.36)	124b (12.68)	1.18b (0.22)	2.21a (0.10)	0.74a (0.05)
		OM	31.4a (1.58)	18.4a (2.47)	10.9b (1.29)	39.3a (1.35)	156a (1.73)	2.91a (0.09)	2.42a (0.04)	0.63ab (0.04)
	20–40	CK	8.4b (1.77)	13.7a (3.75)	60.0a (1.08)	17.8b (1.96)	35b (6.24)	1.39b (0.05)	2.03b (0.07)	0.4b (0.10)
		CF	35.2a (4.27)	5.4a (1.56)	41.4b (2.68)	18.0b (1.28)	83a (9.53)	1.52b (0.04)	2.4a (0.06)	0.86a (0.04)
		OM	41.0a (8.05)	14.5a (5.47)	7.5c (5.62)	36.9a (7.75)	39b (6.49)	2.72a (0.21)	2.21ab (0.10)	0.76a (0.08)
	40–60	CK	25.3ab (1.11)	9.9a (1.74)	41.1ab (5.33)	23.7a (6.20)	17b (3.06)	1.82a (0.25)	2.03b (0.06)	0.72b (0.03)
		CF	17.3b (3.32)	10.9a (0.68)	48.4a (2.54)	23.5a (1.27)	55a (8.57)	1.64a (0.06)	2.38a (0.06)	0.61c (0.04)
		OM	31.7a (3.84)	6.7a (1.01)	29.0b (6.65)	32.6a (5.58)	49a (5.86)	2.14a (0.25)	2.32a (0.02)	0.83a (0.00)

Values in a column followed by the same letter in each soil depth are not significantly different at *P* < 0.05. The standard errors are in parentheses. The abundance of the total nematode is expressed as individuals per 100 g of dry soil. CK, no fertilization; CF, chemical fertilization; OM, organic manure.

Fertilization had a certain effect on the ecological indices in the first-year experiment, and the values of NCR were higher in fertilizer treatments. The MI in OM soil was the highest in a depth of 0–40 cm (*P* < 0.05). Higher values of H′ were shown in CF and OM treatments in soil depth of 20–60 cm. The values of NCR were found higher in CF treatment in 0–40 cm, as well as in OM soil in a depth of 40–60 cm.

### Fertilization affects the microbial abundance and taxonomic composition in different soil depths of jackfruit

The abundances of bacteria and fungi were significantly higher in OM treatment than in CF and CK (Supplementary Tables 3, 4). Richness (Chao1), Shannon and phylogenetic diversity indices confirmed the impact of fertilization on the alpha diversity of both bacterial and fungal communities (Supplementary Figure 1). Bacterial alpha diversity was much lower in all soil depths of OM treatment, compared with CK and CF treatment (*P* < 0.05, Supplementary Figure 1A). The alpha diversity in deep soil (40–60 cm) treated with OM was significantly lower than that in surface soil (0–20 cm). In addition, the chao1 value and phylogenetic diversity indices were much lower in CK and OM treatment (Supplementary Figure 1B), and the Shannon diversity index was significantly lower in CK. The sampling depth in different treatments had a prominent effect on the alpha diversity of fungal communities which showed a higher value in the surface soil.

**FIGURE 1:**
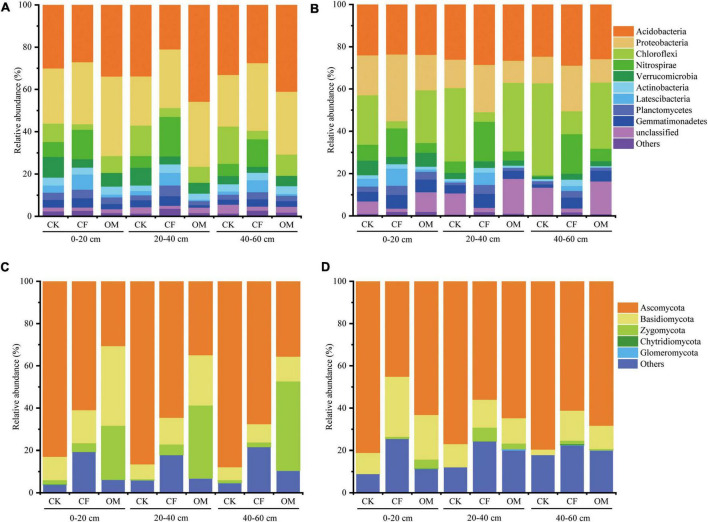
Distribution and abundance of major bacterial **(A,B)** and fungal **(C,D)** phyla in all soil samples with different soil depths. The left column is the samples in the year of 2019, and the right column is in the year of 2020. CK, no fertilization; CF, chemical fertilization; OM, organic manure.

A total of 1,029,274 high-quality bacterial reads and 1,330,571 fungal reads were obtained from 27 soil samples in 2019, and 1,969,457 high-quality bacterial reads and 1,955,677 fungal reads were obtained in 2020. After removing the low-quality and plant-derived reads, the remaining reads were clustered into 10,507 bacterial and 3,670 fungal OTUs in 2019, and 11,045 bacterial and 3,091 fungal OTUs in 2020, respectively. Based on the OTU classification results, Actinobacteria, Proteobacteria, Chloroflexi, and Nitrospirae were the dominant bacterial phyla in treatments with different soil depths, accounting for 70.0–83.9% of the total sequences. Actinobacteria and Proteobacteria dominated the bacterial communities of OM soil in the first-year experiment (Figure 1A). Moreover, Proteobacteria was more prevalent in CF soil in the second-year experiment (Figure 1B). Compared with CF, Chloroflexi was significantly enriched in the CK and OM treatments. Nitrospirae was significantly abundant in CF soil, followed by CK in three soil depths. Ascomycota, Basidiomycota, and Zygomycota were the dominant fungal phyla in treatments with different soil depths, accounting for 74.4–95.8% of the total reads (Figures 1C,D). Ascomycota was significantly enriched in the CK soil. Basidiomycota dominated the OM soil fungal communities in the first-year experiment (Figure 1C) and was more prevalent in CF and OM soils in the second-year experiment (*P* < 0.05, Figure 1D). Zygomycota was significantly abundant in surface soil of OM (0–20 cm) in the 2-year experiments. The variation of phyla in each treatment with different soil depths had similar trends.

**FIGURE 2:**
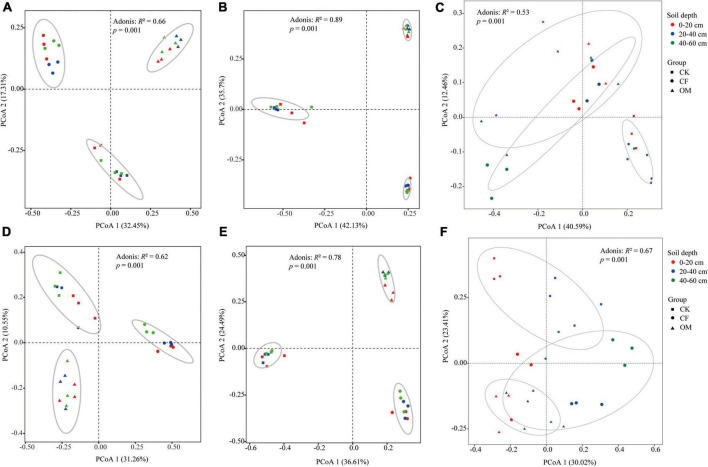
Principal coordinate analysis (PCoA) ordinations of the bacterial **(A,D)**, fungal **(B,E)** and nematode **(C,F)** communities based on Bray–Curtis distance metric in different soil depths. The up-row is the samples in the year of 2019, and the down-row is in the year of 2020. The top value of each plot indicates the fertilization effect on the microbial and nematode communities in analysis of variance (ANOVA) at a significance level of *P* < 0.05. CK, no fertilization; CF, chemical fertilization; OM, organic manure.

Principal coordinate analysis revealed significant differences in the composition of microbial and nematode communities in treatments amended with different fertilizers (Figure 2). Permutational multivariate analysis of the microbial communities was in agreement with PCoAs in that fertilization has a significant impact on microbial and nematode communities in the 2-year experiments. In addition, the difference in microbial community composition explained by fertilization was greater than the difference in nematode community (bacteria: *R*^2^ = 0.66/0.62, *P* = 0.001; fungi: *R*^2^ = 0.89/0.78, *P* = 0.001; nematode: *R*^2^ = 0.29/0.30, *P* = 0.001). In general, the effect of soil depth on microbial and nematode communities was not as significant as that of fertilization (Supplementary Table 5). Therefore, the later analysis was more focused on the effect of fertilization on the microbial and nematode communities as well as SOC components.

### Jackfruit yield, soil physicochemical properties, and their correlation analyses with microbial and nematode communities

Compared with CF and CK, OM treatment resulted in a significant (>13%) increase in jackfruit yield in two consecutive years (Supplementary Tables 3, 4). Soil pH, total nitrogen, phosphorus, potassium concentrations, and SOC components, especially MBC were generally higher in OM treatment in all soil depths in the 2-year field experiments (Supplementary Tables 3, 4). Chemical fertilizers significantly affected AN, AP, and AK concentrations.

**FIGURE 3:**
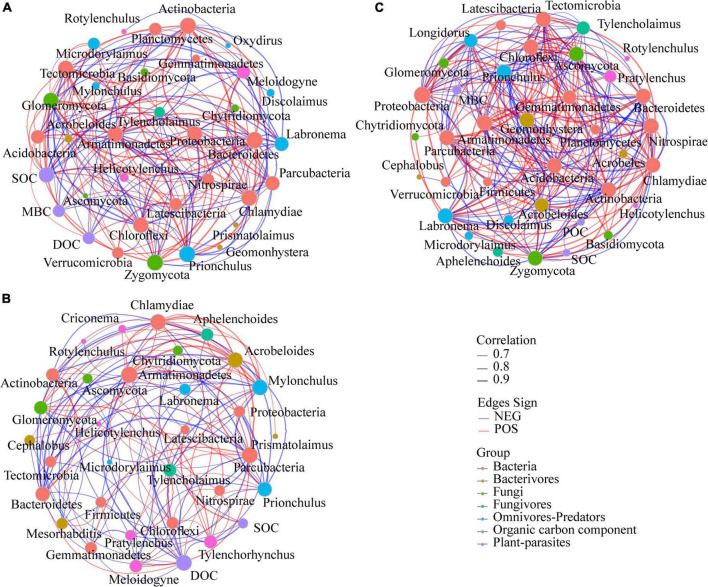
Interaction networks among bacterial and fungal phyla, nematode trophic groups, and SOC components present in different fertilization treatments. A connection stands for a strong (Spearman’s rho > 0.6) and significant (*P* < 0.01) correlation for the CK **(A)**, CF **(B)**, and OM **(C)**. For each panel, the node size is proportional to the number of node connection across all the samples and the thickness of each connection between two nodes (that is, edge) is proportional to the value of Spearman’s correlation coefficients. Lines connecting nodes (edges) represent positive (red) or negative (blue) co-occurrence relationships. CK, no fertilization; CF, chemical fertilization; OM, organic manure.

Network analysis was used to determine the co-occurrence patterns of microbiome, nematode, and SOC components based on strong and significant correlations despite that the calculated modularity index was low (Figure 3 and Supplementary Table 6). Overall, different fertilizer treatments showed a remarkable effect on association networks of nematode and microbiome. The values of average path length (APL) and average clustering coefficient (*avgCC*) in these empirical networks were higher than those of their respective identically sized Erdös–Réyni random networks (Supplementary Table 6). Furthermore, the number of edges, average connectivity (*avgK*), *avgCC* and the percentage of the positive link (P%) of bacteria-fungi were the greatest in OM network, whereas APL was the smallest in CF network. The P% of bacteria-nematode was generally higher in fertilizer treatments, while SOC components showed more positive co-occurrence relationships with microbiota in CK soil. Strikingly, more positive co-occurrence relationships (9.68%) between plant-parasites and others were founded in the soil treated with CF. In addition, the soil of CK treatment showed more negative co-occurrence relationships between plant-parasites *Meloidogyne* and others (SOC, DOC, and the fungi Zygomycota, which were generally lower in CK soil), and more positive co-occurrence relationships between omnivores-Predators *Prionchulus* and SOC components (SOC and DOC). The dominant plant-parasites *Tylenchorhynchus* showed a significantly negative co-occurrence relationship with DOC and a positive co-occurrence relationship with bacteria Nitrospirae which is the preponderant phylum in CF treatment. There were more positive co-occurrence relationships between SOC components and microbial communities in OM soils. The number of links between microbial taxa and microbivorous nematodes in each treatment was OM (29) < CF (24) < CK (4).

**FIGURE 4:**
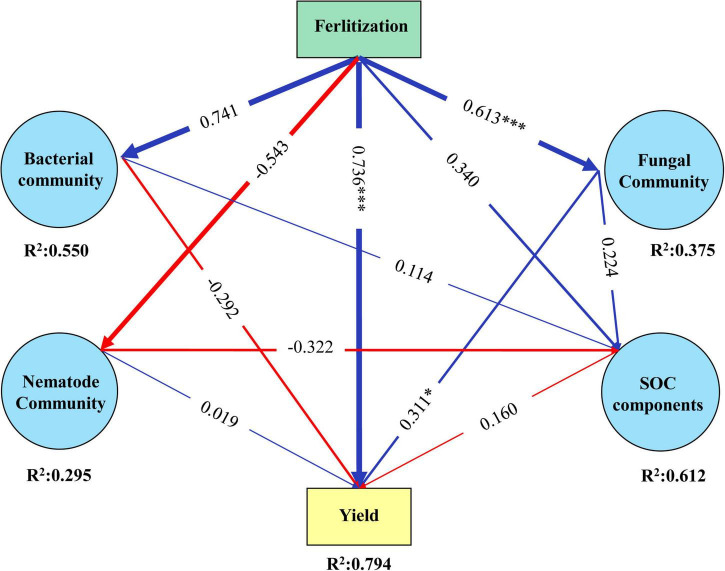
Partial least squares path models (PLS-PM) displaying the direct and indirect effects of long-term fertilization, SOC components (SOC, POC, DOC, and MBC), microbial and nematode community compositions on the jackfruit yield (Goodness-of-Fit = 0.484). Microbial and nematode compositions are used as latent variables, reflecting the relative abundance of each phylum and trophic group, respectively. Coefficient of determination (*R*^2^) values denote the proportion of variance explaining for each variable. Arrow thickness is scaled proportionally to the standardized path coefficients (numbers on arrows). Solid blue and red arrows indicate positive and negative relationships, respectively.

As mentioned above, the correlation analysis displays the possible relationship of microbial and nematode communities with SOC components. However, it cannot uncover the direct (or indirect) causal relationship. Partial least-squares-path modeling was employed to quantify the specific causality from a holistic view. As can be seen, the model explained 79.4% of the variation in jackfruit yield (*R*^2^ = 0.794, Figure 4). Fertilization had significant effects on jackfruit yield, which was composed of positive direct (73.6%) and indirect effects (fertilization → fungal community → yield). Likewise, fertilization induced changes in soil microbial and nematode communities imposed indirect effects on SOC components and jackfruit yield despite that their effect was not significant.

### Functional prediction analysis

**FIGURE 5:**
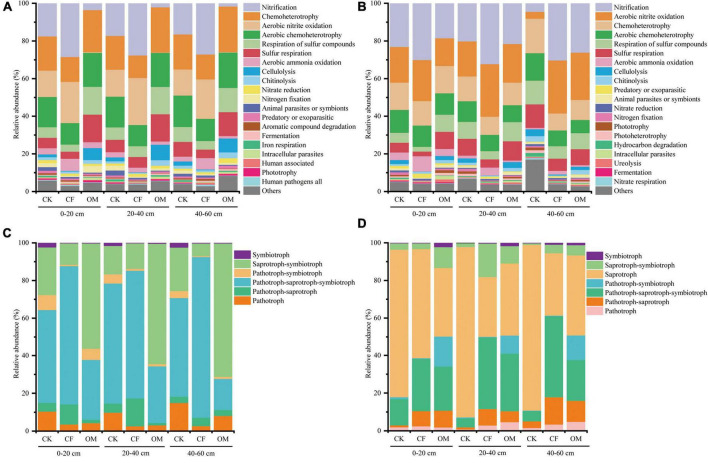
The relative abundance of dominant bacterial **(A,B)** and fungal **(C,D)** functional groups based on Functional Annotation of Prokaryotic Taxa (FAPROTAX) and FUNGuild. The left column is the samples in the year of 2019, and right column is in the year of 2020. CK, no fertilization; CF, chemical fertilization; OM, organic manure.

Functional Annotation of Prokaryotic Taxa analysis was performed for the determination of predicted functions of bacterial communities in different fertilizer treatments in jackfruit orchards. Six ecological function groups related to the C cycles, including aerobic chemoheterotrophy, chemoheterotrophy, cellulolysis, phototrophy, photoheterotrophy, and aromatic compound degradation, accounted for an average of 32% of the total abundance of predictive functional analysis (Figures 5A,B). The proportions of chemoheterotrophy, aerobic chemoheterotrophy and cellulolysis in the OM soils were significantly higher than those observed in other treatments in the first year while this trend abated in the second year. Six ecological function groups related to the N cycles were classified as aerobic ammonia oxidation, nitrification, aerobic nitrite oxidation, nitrate reduction, nitrogen fixation, and nitrogen respiration, accounting for an average of 41% of the total abundance of predictive functional analysis in our study. The relative abundance of nitrification, aerobic nitrite oxidation and aerobic ammonia oxidation identified in the CF soils were significantly higher than those observed in the other treatments.

FUNGuild was used to predict the nutritional and functional groups of fungi, and the results showed that symbiotroph, saprotroph-symbiotroph, saprotroph, pathotroph-symbiotroph, pathotroph- saprotroph-sym-biotroph, pathotroph-saprotroph, and pathotroph were the major components (Figures 5C,D). The proportions of saprotroph-symbiotroph in the OM soils were significantly higher than those observed in the other treatments in the first-year experiment. On the contrary, the relative abundance of pathotroph-saprotroph and pathotroph-saprotroph-symbiotroph in soil depth of 0–40 cm of CF treatment was significantly higher compared with OM treatment.

## Discussion

### Effects of fertilization on soil nematode community

The treatments of CF and OM are representative of soil management systems commonly used in jackfruit orchards. The carbon and energy inputs to the soil food web can generally be delivered along with the trophic levels and affect the abundances of total nematodes and trophic groups present at different trophic levels (Chen et al., 2021). In this study, OM had significantly positive effects on the abundances of total nematodes and trophic groups, except for plant-parasites (Table 1). This is consistent with the findings by Liu et al. (2016) that integrated 54 relevant studies around the world and showed that organic amendment input improved soil nematode abundance by 37–50%. In addition, manure is more labile for microorganism decomposition which provided more energy and carbon to the nematode assemblage, and more nutrients were released after manure decomposition (Elzobair et al., 2016; Liu et al., 2020). This explains a higher percentage of bacterivores that was found in the manure amendment. Carrillo et al. (2011) also reported a similar finding that microbivorous nematodes positively affected microbial activity during decomposition. As the response of soil nematode was linked to the soil microbial biomass, soil MBC has been found highly correlated with the nematode beta diversity and community in this study (Figure 3). As the main decomposers in the soil, microbes first metabolize organic matter and then transfer energy and carbon to higher trophic groups, including nematodes (Liu et al., 2020). Therefore, soil MBC content and microbivores were increased in OM treatment compared with CK and CF treatments. Organic manure amendments strongly stimulated the basal functional guilds of the nematode community, as indicated by the high populations of c-p 1 (*Mesorhabditis*) and 2 (*Cephalobus*, *Geomonhystera*, and *Acrobeloides*) bacterivores. The current study showed that the predominant trophic group under CK and CF treatments was plant-parasites, particularly *Pratylenchus*, *Rotylenchulus*, and *Meloidogyne*, which occupied more than 33.6 and 41.4% of the total nematode abundance in the 2-year field experiments, respectively. Liu et al. (2013) also reported that the sole application of mineral fertilizer decreased the physiological resistance of the crop, and the weaker roots would have been easily infected by insects such as plant-parasites. In contrast, previous studies have reported that manure application provides carbon for soil organisms and improves plant resistance, thereby, increasing soil bacteria abundance and leading to a transition in the predominant trophic group from plant-parasites to bacterivores (Wu et al., 2016). The higher abundance of nematodes occurred in the 0–20 cm depth, where most roots are distributed and soil has better aerobic conditions, which creates a better environment for the survival of nematodes in this layer (Van Nguyen et al., 2020).

The nematode community structure, decomposition environments, and the dynamics of soil food webs could be evaluated by the community indices. In the present study, we observed that OM increased the MI value in soils with a depth of 0–40 cm, indicating that manure amendment drives the soil food web toward a relatively stable environment for crop productivity (Table 1). Higher H′ in the organic mature treatment indicates that the nematode community was more diverse and that some genera dominated the community. As a result of the higher NCR values in fertilizer treatments, bacteria appear to dominate the organic matter decomposition pathway.

### Effects of fertilization on soil microbial community and functional groups

In the present study, the microbial communities in soils that received long-term manure amendment presented significantly higher microbial abundance than those receiving CFs in both layers, which may have been due to the higher soil pH. A previous study also showed that the increased pH may enhance the spore germination, colonization, and reproduction rates of microbes and consequently increased microbial biomass (Chen et al., 2019). Fertilization with OM typically alters the soil microflora, such as richness, diversity, and community composition (Chen et al., 2019). Our results also supported this finding that both soil microbial α-diversity and community composition were significantly changed by OM treatment. Soil bacterial richness and phylogenetic diversity immediately decreased in OM treatment in the first-year experiment, but they rebounded to greater values in the second-year experiment. These findings were probably related to the higher relative abundances of major microbial groups (Acidobacteria and Proteobacteria) found in the soil (St-Pierre and Wright, 2014). Changes in the soil fungal α-diversity may be similar.

The bacterial phylum Proteobacteria (Gamma- or Beta-) which was considered a copiotrophic group and related to C availability or labile substrate supply was one of the predominant taxa in manure amendment in both layers, which may have been due to the rich nutrient in the soil (Liu et al., 2019). Most members of Acidobacteria and Chloroflexi have been identified as oligotrophic groups (Fierer et al., 2007), and our study showed higher relative abundances of the Acidobacteria and Chloroflexi phyla in OM and CK soil than in the CF soil. This result was inconsistent with the results of many previous studies and it might relate to the diversely nutritional profile of Chloroflexi which can change depending on the environmental conditions. The higher relative abundance of Nitrospira in CF than other treatments indicates that Nitrospira, and potentially nitrification, was of greater importance in the CF soil, which might be driven by greatly AN release from CF (Supplementary Tables 3, 4). In addition, some Nitrospirae strains can be a dominant nitrite oxidizer or comammox which convert urea to ammonia and CO_2_ and may contribute to nitrogen cycling beyond nitrite oxidation (Wang et al., 2019). In our study, compared with other treatments, the functional groups of the bacterial community related to C cycling (e.g., chemoheterotrophy, aerobic chemoheterotrophy, and cellulolysis) were higher in OM soil, which might be due to the addition of OM. The variation of plant root exudates can directly provide a large number of carbon sources and promote the assimilation, utilization of carbon by microorganisms, thus promoting the increase of chemical heterotrophic microorganisms (Liang et al., 2020). Different vegetation types could cause changes in bacterial community function in the soil. Conversely, nitrification, aerobic nitrite oxidation, and aerobic ammonia oxidation related to the nitrogen cycle increased in the CF soils. The reason for this difference might be due to the increased nitrogen content affected by CF, which stimulated the growth and reproduction of denitrifying microorganisms, significantly increased microbial activity, and altered N cycles (Liang et al., 2020).

In the current study, Ascomycota, Basidiomycota, and Zygomycota were the dominant phyla of fungal community (Figures 1C,D). In alignment with a previous study by Ji et al. (2020), Ascomycota and Zygomycota exhibited different growth strategies with organic fertilizer application, and Zygomycota saprotroph exhibited increased sensitivity to C sources than the Ascomycota saprotroph, which consequently resulted in a different relative abundance of Ascomycota and Zygomycota in manure amendment. Moreover, Štursová et al. (2012) found that Zygomycota was an important decomposer for controlling the cycling rate of nutrients and promoting the decomposition of organic compound matrices in agricultural ecosystems. Seven fungal functional groups (i.e., symbiotroph, saprotroph-symbiotroph, saprotroph, pathotroph-symbiotroph, pathotroph-saprotroph-symbiotroph, pathotroph-saprotroph, and pathotroph) were identified according to FUNGuild (Guo et al., 2020). Our results found that saprotroph-symbiotroph fungi were the dominant function fungi and accounted for more than 50% of the whole community under OM treatment in the first-year experiment. In contrast, pathotroph-saprotroph-symbiotroph and pathotroph-saprotroph fungi predominated in CF treatment (Figure 4), which indicated a risk of plant disease (Ji et al., 2020).

### Effects of fertilization on β-diversity of soil microbe and nematode

As for β-diversity of microbe and nematode, irrespective of the soil layers, all soil samples were clustered into three groups according to fertilizer treatments (Figure 2), which suggested that differential fertilization was the dominant factor in shaping the microbial-microfauna in the soil of jackfruit. Variations in β-diversity can be attributed to fertilizer, since shifts in soil microbial communities generally correlate with changes in soil nutrient availability (Zhang et al., 2018). In addition, the variation of nematode fauna was closely related to the microbial community structure.

### Effects of fertilization on jackfruit yield and soil physicochemical properties

The extra resource input either chemical or organic fertilizer enhanced jackfruit yield compared with no input control during the 2-year field experiments. Average jackfruit yield increased by 15% in the sole CF treatments and by 32% in the manure amendment relative to the control (Supplementary Tables 3, 4). This result reiterates the necessity for fertilizer additions to increase crop yields. Organic manure could enhance jackfruit yield not only through the continuous supply of reserve nutrients but also as a result of better soil conditions for crop growth such as soil aeration, porosity, soil pH, and microflora (Cai et al., 2019). In our study, soil properties also indicated fertilization inputs influenced nutrient availability (Supplementary Tables 3, 4). Soil pH in both layers was found to be lower in CF treatment which might be due to the produced H^+^ ions during the nitrification of NH_4_^+^ (Luo et al., 2015). While, the addition of manure prevented soil acidification due to the alkalinity of manure (Rukshana et al., 2014). Organic manure significantly increased soil total nutrient contents and organic carbon components, especially MBC, compared with chemical fertilization alone. This can be confirmed by Tian et al. (2015), who reported that continuous manure application increased the SOC content and sequestration rates by increasing crop yield and organic matter return from stubbles and roots in a meta-analysis study. In addition, the rate of soil mineralization usually remains stable for a short time, so the increasing trend of SOC content in soils could be explained by the C input from organic amendments (Li et al., 2020). The increased soil MBC in the OM treatment may be due to the additional C sources, which are beneficial for the growth of soil indigenous microbiota as well as an increase in soil fertility (Li et al., 2015). In the subsoil layers (40–60 cm), the SOC contents in all treatments were lower than in the top layers. This decrease could be explained by the possible presence of roots and an abundance of microorganisms in the Ap horizon (near the soil surface) (Shahbaz et al., 2017). Labile C (e.g., DOC, MBC, and POC) is sensitive to fertilization management and a good indicator to study SOC changes on a short-term basis. In the present study, the application of OM had a positive effect on LOC in both layers. LOC in subsurface soil was much lower as compared to surface soil, which might be attributed to an increase in the recalcitrant fraction of C in subsurface soil (Ghosh et al., 2012).

### Correlations between microbial and nematode communities and organic carbon components

Network analyses and PLS-PM also indicated that fertilization induced soil microbial and nematode communities, and then indirectly influenced SOC components. Significant co-occurrence relationships between soil microflora and organic carbon components have been observed in the present study. The soil amended with OM showed a higher number of positive co-occurrence relationships than that in other treatments which may be linked to a higher community function (Coyte et al., 2015). And the soils amended with CF and nothing contained more strongly co-occurrence relationships between plant-parasites and others which suggesting that the CF and CK treatments may increase the abundance of plant-parasites and microorganisms associated with them. In the contrast, OM may be associated with a decreased ratio of positive link of plant-parasites. Previous studies had shown that the feeding interrelationship among the soil biota had a strong influence on the flow of resource and energy within the soil food web (Lenoir et al., 2007; Zhang X. et al., 2015). The more co-occurrence relationships between microbivorous nematodes and microbial taxa that had positive associations with SOC components in OM soils were also supported by other studies that have reported that the predation by microbivorous nematodes changed the microbial communities that are responsible for the breakdown of organic matter were linked to soil organic matter decomposition (Freckman, 1988; Jiang et al., 2018). In agroecosystems, nutrient release and dissolved C during long-term decomposition of fertilizer directly affected soil microbial community composition (Diacono and Montemurro, 2010). The variation of this soil property (e.g., soil C, pH) resulted from fertilization exerts significant influences on microbial growth (Hartmann et al., 2015; Sun et al., 2016; Zeng et al., 2016; Zhang et al., 2017). In the current study, a greater addition of OM increased SOC components and pH; these changes increased the niche width and niche differentiation (Dumbrell et al., 2010), and these factors may be important to the diversity with beneficial coexistence of species in the soil habitat. An oligotroph-copiotroph strategy shift of soil bacteria with changes in soil nutrient availability has been observed by Fierer et al. (2012), who reported that low nutrient levels caused an increase in slow-growing oligotrophic microorganisms while high nutrient levels promoted copiotrophic organisms. Differential soil properties affected by fertilizer amendments might exert a direct or indirect influence on nematode fauna *via* plant growth or microbial activity (Bulluck and Ristaino, 2002; Buchan et al., 2013). These results were most likely due to the increased C, N, P, K, and pH of soil by OM, and these factors may be important drivers of soil microfauna and crop yield.

## Conclusion

An assessment was carried out on the impacts of fertilization amendments on selected soil physicochemical properties and microbial and nematode communities in three soil depths (0–20, 20–40, and 40–60 cm) over an 8-year period in a mono-cultured jackfruit plantation. In general, OM increased the value of NCR, MI, and the abundance of total nematodes and bacterivores, but decreased plant-parasites and H′. The microbial β-diversity and taxonomical composition showed a distinct response to the applied treatments, especially at the phyla level. Higher relative abundances of Proteobacteria, Acidobacteria, and Chloroflexi were observed in OM treatment, and Nitrospira was predominated in CF treatment. Furthermore, OM enhances the contents of soil N, P, K, C, and pH, and the variation of this soil property was an important driver of soil microbial and nematode communities, functional groups and crop yield. Our results indicated that the functional groups of the bacterial community related to C cycle and aprotroph-symbiotroph fungi were higher in OM soil, while, some groups related to the nitrogen cycle, pathotroph-saprotroph-symbiotroph and pathotroph-saprotroph were predominated in CF treatment. This research may be beneficial in improving the understanding of the relationship between fertilization amendment, soil quality, and soil microbial and nematode communities, which can contribute to the development of an effective nutrient management system toward sustainability. In future studies, soil microfauna and the functional group should be complemented with the responses of crop roots to enhance our understanding of the mechanisms by which manure affects the soil quality for crop production.

## Data availability statement

The datasets presented in this study can be found in online repositories. The names of the repository/repositories and accession number(s) can be found below: NCBI–PRJNA836735 and the release date was June 5, 2024.

## Author contributions

LS: conceptualization, development of methodology, performing the experiments, data analysis, and writing original manuscript. TB and YX: conceptualization, methodology, writing – review and editing, and supervision. GW: supervision and writing – review and editing. QZ: methodology. LT: conceptualization, writing – review and editing, and supervision. All authors have read and approved the final manuscript and approved the submitted version.

## Abbreviations

CK: no fertilization
CF: chemical fertilization
OM: organic manure
SOC: soil organic carbon
MBC: microbial biomass carbon
POC: potassium permanganate-oxidizable carbon
DOC: dissolved organic carbon.
