# Supplementary material for: Characteristics of soil microbiota and organic carbon distribution in jackfruit plantation under different fertilization regimes
Source: Front Microbiol. 2022 Sep 20;13:980169. doi: 10.3389/fmicb.2022.980169 (PMC9530185; doi:10.3389/fmicb.2022.980169)
Supplement: Supplementary file 1 [file Data_Sheet_1.docx]

**Supplementary Figures and Tables**

**Characteristics of soil microbiota and organic carbon distribution in jackfruit plantation under different fertilization regimes**

Lanxi Su^1,2,3^, Tingyu Bai^1,2,3^, Gang Wu^1,2,3^, Qingyun Zhao^1,3^, Lehe Tan^1,2^*, Yadong Xu^4^**

Affiliations

^1^ Spice and Beverage Research Institute, Chinese Academy of Tropical Agricultural Sciences, Wanning, Hainan, 571533, China.

^2^ National Tropical Plants Germplasm Resource Center-Sub Centre of Germplasm Resource for woody grain, Wanning, Hainan, 571533, China.

^3^ Key Laboratory of Genetic Improvement and Quality Regulation for Tropical Spice and Beverage Crops of Hainan Province, Wanning, Hainan, 571533, China.

^4^ School of Agricultural Sciences, Zhengzhou University, Zhengzhou, 450001, China


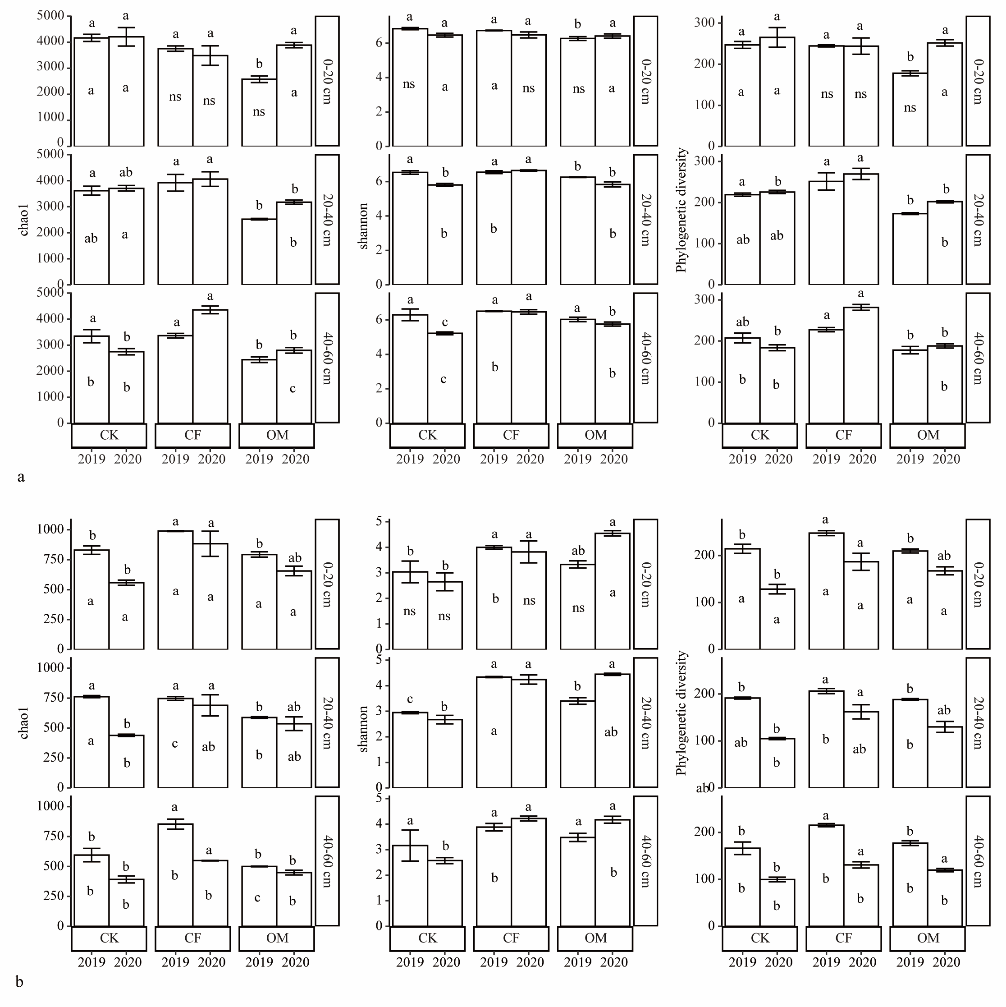


Fig.S.1 Richness and phylogenetic diversity indices of bacteria (a) and fungi (b) in all soil samples with different soil depths.

Different letters above bars in each year mean significantly different among fertilization treatments at *P* < 0.05, and the [letter](javascript:;)s ‘ns’ in the bars mean no significant difference among soil depth at *P* < 0.05. CK, no fertilization; CF, chemical fertilization; OM, organic manure.

**Table S.1** Nematode abundance (ind./100 g dry weight soil) of individual taxa in all soil samples with different soil depths in 2019.

| Trophic Group | Nematode taxa | 0-20 cm | | | 20-40 cm | | | 40-60 cm | | |
| --- | --- | --- | --- | --- | --- | --- | --- | --- | --- | --- |
|  |  | CK | CF | OM | CK | CF | OM | CK | CF | OM |
| Bacterivores | *Geomonhystera* | 1.0a (1.00) | 1.0a (1.00) | 2.0a (1.15) | 1.3a (1.33) | 2.3a (1.45) | 3.3a(0.88) | 0.0a(0.00) | 0.0a(0.00) | 0.0a(0.00) |
|  | *Cephalobus* | 4.0b (1.15) | 3.7b (0.67) | 8.7a (0.88) | 10.0ab (5.03) | 2.0b (0.00) | 18.7a(2.91) | 1.7b(0.67) | 5.7ab(1.33) | 16.7a(5.49) |
|  | *Mesorhabditis* | 4.0b (0.58) | 5.7b (0.33) | 16.3a (4.91) | 13.3a (8.35) | 2.3a (0.33) | 16.0a (4.04) | 1.7b (0.67) | 11.7a (2.33) | 6.0ab (3.51) |
|  | *Prismatolaimus* | 1.7a (0.88) | 0.0a (0.00) | 14.7a (7.84) | 0.0b (0.00) | 0.0b (0.00) | 6.0a (1.53) | 0.0b (0.00) | 2.3ab (2.33) | 6.3a (1.86) |
|  | *Acrobeles* | 0.0a (0.00) | 0.0a (0.00) | 8.3a (4.48) | 0.0a (0.00) | 0.0a (0.00) | 0.0a (0.00) | 0.0a (0.00) | 0.0a (0.00) | 0.0a (0.00) |
|  | *Acrobeloides* | 1.7a (0.88) | 0.0a (0.00) | 2.0a (1.15) | 0.0b (0.00) | 0.0b (0.00) | 5.3a (2.03) | 0.0a (0.00) | 0.0a (0.00) | 0.0a (0.00) |
| Fungivores | *Tylencholaimus* | 24.0ab (2.89) | 14.3b (4.91) | 32.0a (5.13) | 9.7b (2.03) | 3.0b (1.00) | 29.3a (6.06) | 5.3b (1.33) | 11.3b (4.33) | 32.0a (2.00) |
|  | *Aphelenchoides* | 1.7a (1.67) | 1.0a (1.00) | 0.0a (0.00) | 1.3a (1.33) | 0.0a (0.00) | 0.0a (0.00) | 0.7a (0.33) | 0.0a (0.00) | 0.0a (0.00) |
| Plant-parasites | *Pratylenchus* | 8.3a (2.33) | 18.0a (4.93) | 22.3a (7.06) | 18.3ab (4.18) | 3.0b (1.00) | 41.3a (12.71) | 4.3a (0.88) | 20.0a (4.04) | 29.3a (11.35) |
|  | *Rotylenchulus* | 8.3a (1.86) | 10.3a (4.33) | 2.7a (1.76) | 6.3a (3.18) | 4.0a (1.15) | 0.0a (0.00) | 1.7b (1.20) | 13.7a (2.03) | 1.3b (1.33) |
|  | *Meloidogyne* | 3.0a (0.58) | 9.3a (4.91) | 4.0a (2.65) | 0.0a (0.00) | 3.0a (1.00) | 7.0a (3.61) | 0.3b (0.33) | 5.7ab (1.33) | 10.3a (2.67) |
|  | *Helicotylenchus* | 6.0a (1.53) | 4.7a (0.88) | 2.7a (1.33) | 3.3a (1.76) | 4.7a (2.19) | 0.0a (0.00) | 0.3a (0.33) | 1.0a (1.00) | 0.0a (0.00) |
|  | *Tylenchorhynchus* | 1.3a (1.33) | 0.0a (0.00) | 6.0a (5.03) | 5.0a (2.65) | 1.3a (0.67) | 3.0a (1.73) | 1.7a (1.20) | 0.0a (0.00) | 10.7a (5.61) |
|  | *Criconema* | 3.0a (1.53) | 0.0a (0.00) | 1.0a (1.00) | 0.0a (0.00) | 0.0a (0.00) | 0.7a (0.67) | 0.0a (0.00) | 0.0a (0.00) | 0.0a (0.00) |
| Omnivores-Predators | *Oxydirus* | 5.0a (0.58) | 1.0b (1.00) | 2.3ab (1.20) | 5.0a (2.65) | 1.0a (1.00) | 1.3a (0.67) | 1.0a (0.00) | 4.7a (2.33) | 4.0a (0.58) |
|  | *Prionchulus* | 8.0a (4.51) | 10.7a (0.67) | 8.3a (2.91) | 9.7a (2.03) | 1.3b (0.67) | 3.0b (1.00) | 3.3a (1.20) | 5.7a (1.33) | 6.3a (1.86) |
|  | *Labronema* | 0.0a (0.00) | 3.0a (1.73) | 0.0a (0.00) | 0.0a (0.00) | 0.0a (0.00) | 0.0a (0.00) | 0.0a (0.00) | 0.0a (0.00) | 0.0a (0.00) |
|  | *Mylonchulus* | 1.0a (1.00) | 0.0a (0.00) | 3.3a (1.76) | 5.0a (2.65) | 1.3a (0.67) | 3.0a (0.58) | 0.0a (0.00) | 0.0a (0.00) | 2.0a (1.15) |
|  | *Discolaimus* | 0.7a (0.67) | 0.0a (0.00) | 2.0a (1.15) | 2.0a (2.00 ) | 0.0a (0.00) | 0.0a (0.00) | 0.0a (0.00) | 0.0a (0.00) | 2.7a (1.45) |
|  | *Microdorylaimus* | 6.7a (1.76) | 6.0a (2.08) | 6.3a (1.45) | 5.0a (2.65) | 2.3a (0.33) | 3.3a (0.88) | 0.7b (0.33) | 0.0b (0.00) | 4.0a (0.58) |

Values in a row followed by the same letter in each soil depth are not significantly different at *P* < 0.05. The standard errors are in parentheses. CK, no fertilization; CF, chemical fertilization; OM, organic manure.

**Table S.2** Effect of organic manure and chemical fertilization treatments on nematode abundance (individuals 100 g^-1^) of individual taxa in soil samples with different soil depths in 2020.

| Trophic Group | Nematode taxa | 0-20 cm | | | 20-40 cm | | | 40-60 cm | | |
| --- | --- | --- | --- | --- | --- | --- | --- | --- | --- | --- |
|  |  | CK | CF | OM | CK | CF | OM | CK | CF | OM |
| Bacterivores | *Geomonhystera* | 0.3b (5.17) | 2.7b (12.68) | 7.7a (1.73) | 0.0b (6.24) | 3.7a (9.53) | 3.0a (6.49) | 0.0b (3.06) | 0.7b (8.57) | 2.7a (5.86) |
|  | *Cephalobus* | 2.3b (0.33) | 6.7a (0.33) | 3.0ab (1.45) | 2.0b (0.00) | 13.3a (0.88) | 3.0b (1.15) | 1.0a (0.00) | 6.0a (0.67) | 3.7a (0.67) |
|  | *Mesorhabditis* | 2.0b (0.88) | 3.3b (0.67) | 9.3a (1.53) | 0.3b (0.58) | 6.3a (3.28) | 6.7a (1.53) | 2.0a (0.58) | 0.0b (1.53) | 3.7a (1.86) |
|  | *Prismatolaimus* | 1.3b (1.15) | 2b (0.88) | 9.3a (2.33) | 0.0b (0.33) | 3.0a (2.19) | 0.7b (1.20) | 0.0a (0.00) | 0.7a (0.00) | 1.3a (0.88) |
|  | *Acrobeloides* | 2.3b (0.67) | 4.3b (1.00) | 19.7a (2.33) | 0.7a (0.00) | 3.0a (0.58) | 3.0a (0.67) | 1.3b (0.00) | 2.7ab (0.67) | 4.3a (0.67) |
| Fungivores | *Tylencholaimus* | 5.0b (0.33) | 4.7b (1.86) | 23.7a (3.76) | 4.0a (0.33) | 2.3a (1.00) | 4.7a (1.53) | 1.7a (0.67) | 4.0a (0.67) | 2.7a (0.33) |
|  | *Aphelenchoides* | 1.0a (1.15) | 2.3a (0.88) | 5.0a (2.67) | 1.0a (1.53) | 2.3a (1.45) | 1.7a (1.86) | 0.0b (0.33) | 2.0a (1.15) | 0.7b (0.67) |
| Plant-parasites | *Pratylenchus* | 10.7a (0.58) | 3.0a (1.45) | 8.3a (2.89) | 3.7ab (0.58) | 6.3a (1.20) | 0.3b (0.88) | 1.7a (0.00) | 5.3a (0.00) | 5.0a (0.67) |
|  | *Rotylenchulus* | 1.3b (0.88) | 15.3a (1.73) | 0.0b (4.41) | 7.0a (0.88) | 3.7ab (2.19) | 0.0b (0.33) | 1.3a (0.33) | 2.7a (0.67) | 0.7a (1.73) |
|  | *Meloidogyne* | 28.3ab (1.33) | 34.3a (4.10) | 7.3b (0.00) | 8.0b (2.65) | 15.7a (0.88) | 1.7c (0.00) | 3.0a (0.88) | 8.7a (0.67) | 9.0a (0.67) |
|  | *Helicotylenchus* | 0.0a (9.84) | 19.0a (3.53) | 0.0a (5.04) | 0.0a (0.00) | 1.7a (2.19) | 0.0a (0.88) | 0.7ab (0.58) | 3.0a (1.20) | 0.0b (4.04) |
|  | *Tylenchorhynchus* | 12.7a (0.00) | 2.7ab (10.6) | 1.3b (0.00) | 2.0b (0.00) | 6.0a (0.88) | 0.3b (0.00) | 0.0b (0.67) | 5.0a (1.00) | 0.0b (0.00) |
|  | *Criconema* | 0.7a (4.84) | 3.0a (1.45) | 0.0a (1.33) | 0.3a (0.58) | 0.7a (1.53) | 0.0a (0.33) | 0.0a (0.00) | 1.3a (0.58) | 0.0a (0.00) |
| Omnivores-Predators | *Oxydirus* | 6.0a (0.67) | 0.0b (1.73) | 9.3a (0.00) | 0.0a (0.33) | 0.0a (0.67) | 1.0a (0.00) | 1.3a (0.00) | 0.0a (0.67) | 0.0a (0.00) |
|  | *Prionchulus* | 0.7a (1.53) | 0.0a (0.00) | 4.3a (2.33) | 0.7a (0.00) | 0.0a (0.00) | 1.0a (0.58) | 0.0b (0.88) | 0.0b (0.00) | 2.7a (0.00) |
|  | *Labronema* | 5.7b (0.67) | 7.7b (0.00) | 23.7a (2.33) | 4.3a (0.67) | 5.0a (0.00) | 7.7a (0.58) | 2.7b (0.00) | 3.3b (0.00) | 7.3a (0.67) |
|  | *Mylonchulus* | 5.7a (2.40) | 9.7a (4.33) | 3.3a (1.86) | 0.3b (2.19) | 8.0a (1.15) | 0.0b (1.67) | 0b (1.20) | 8.7a (0.67) | 1.3b (0.88) |
|  | *Discolaimus* | 3.0ab (3.67) | 0.0b (0.67) | 6.3a (1.67) | 0.7a (0.33) | 0.7a (1.00) | 3.0a (0.00) | 0.3a (0.00) | 0.0a (2.19) | 1.3a (0.67) |
|  | *Microdorylaimus* | 2.3b (0.58) | 3.7b (0.00) | 14.3a (1.86) | 0.0a (0.67) | 1.0a (0.67) | 1.7a (1.15) | 0.0b (0.33) | 0.7b (0.00) | 2.7a (0.67) |

Values in a row followed by the same letter in each soil depth are not significantly different at *P* < 0.05. Standard error is in parentheses. CK, no fertilization; CF, chemical fertilization; OM, organic manure.

**Table S.3** Effect of organic manure and chemical fertilization treatments on soil properties with different soil depths in 2019.

| Soil depth (cm) | Treatment | Soil pH | SOC  g/kg | DOC  mg/g | POC  mg/g | MBC  mg/kg | TN  g/kg | TP  g/kg | TK  g/kg | AN  mg/kg | AP  mg/kg | AK  mg/kg | Bacterial abundance | Fungal abundance | Yield  Mg ha^-1^ |
| --- | --- | --- | --- | --- | --- | --- | --- | --- | --- | --- | --- | --- | --- | --- | --- |
| 0-20 cm | CK | 5.67b (0.03) | 13.21b (0.16) | 0.26b (0.03) | 5.45b (0.11) | 32.62c (0.24) | 0.66b (0.01) | 0.87c (0.00) | 0.16b (0.01) | 53.64a (1.91) | 269.80b (1.00) | 38.36c (1.60) | 8.46c (0.10) | 7.13c (0.05) | 30.28c (0.44) |
|  | CF | 5.10c (0.01) | 12.38c (0.08) | 0.32b (0.02) | 5.68b (0.09) | 63.86b (1.24) | 0.71b (0.06) | 1.45b (0.01) | 0.65a (0.01) | 55.23a (1.41) | 279.77a (1.52) | 160.85a (1.84) | 9.03b (0.05) | 7.95b (0.05) | 34.73b (0.61) |
|  | OM | 6.51a (0.02) | 15.93a (0.14) | 0.42a (0.01) | 10.30a (0.07) | 121.57a (2.22) | 1.00a (0.03) | 2.85a (0.02) | 0.67a (0.02) | 56.82a (1.41) | 180.28c (2.08) | 67.23b (1.65) | 10.10a (0.13) | 8.75a (0.04) | 39.34a (0.30) |
| 20-40 cm | CK | 4.74b (0.07) | 13.26a (0.13) | 0.33a (0.02) | 4.45b (0.04) | 45.69a (1.60) | 0.60c (0.03) | 0.85b (0.01) | 0.16c (0.01) | 49.39b (1.84) | 31.56c (1.27) | 23.10c (0.47) | 7.96c (0.08) | 7.02c (0.07) |  |
|  | CF | 4.91b (0.03) | 10.95b (0.35) | 0.32a (0.01) | 5.48a (0.13) | 29.97b (1.77) | 0.84b (0.02) | 0.89b (0.02) | 0.35b (0.01) | 46.20b (1.84) | 55.77b (0.72) | 117.77a (0.96) | 8.84b (0.05) | 7.55b (0.03) |  |
|  | OM | 6.24a (0.06) | 12.85a (0.14) | 0.30a (0.03) | 5.31a (0.15) | 44.82a (2.02) | 0.93a (0.01) | 1.43a (0.02) | 0.74a (0.01) | 55.76a (1.59) | 154.85a (5.91) | 61.73b (2.16) | 9.76a (0.05) | 8.01a (0.07) |  |
| 40-60 cm | CK | 4.90b (0.02) | 13.77a (0.13) | 0.33a (0.02) | 2.36c (0.24) | 33.22c (0.59) | 0.58b (0.01) | 0.63c (0.02) | 0.20c (0.01) | 43.02b (1.84) | 53.28c (1.48) | 72.01b (1.14) | 7.43c (0.04) | 6.78c (0.05) |  |
|  | CF | 4.83b (0.03) | 8.58c (0.21) | 0.35a (0.02) | 4.18b (0.04) | 61.84b (0.38) | 0.58b (0.01) | 1.11b (0.02) | 0.43b (0.01) | 63.73a (0.92) | 200.40a (2.12) | 95.18a (0.51) | 8.32b (0.07) | 7.26b (0.07) |  |
|  | OM | 6.87a (0.03) | 11.85b (0.21) | 0.36a (0.02) | 10.45a (0.45) | 92.89a (1.84) | 0.72a (0.01) | 1.39a (0.01) | 0.83a (0.02) | 45.14b (1.41) | 111.79b (5.56) | 50.40c (1.30) | 8.89a (0.07) | 7.85a (0.05) |  |

Values in a column followed by the same letter in each soil depth are not significantly different at *P* < 0.05. Standard error is in parentheses. Bacterial or Fungal copy numbers were log10-transformed in abundance. SOC, soil organic carbon; DOC, dissolved organic carbon; POC, potassium permanganate-oxidizable carbon; MBC, microbial biomass carbon; TN, total soil nitrogen; AN, alkalyzable nitrogen; TP, Total phosphorus concentration; AP, available phosphorus; TK, total potassium concentration; AK, readily available potassium (the same below). CK, no fertilization; CF, chemical fertilization; OM, organic manure.

**Table S.4** Effect of organic manure and chemical fertilization treatments on soil properties with different soil depths in 2020.

| Soil depth (cm) | Treatment | pH | SOC  g/kg | DOC  mg/g | POC  mg/g | MBC  mg/kg | TN  g/kg | TP  g/kg | TK  g/kg | AN  mg/kg | AP  mg/kg | AK  mg/kg | Bacterial abundance | Fungal abundance | Yield  Mg ha^-1^ |
| --- | --- | --- | --- | --- | --- | --- | --- | --- | --- | --- | --- | --- | --- | --- | --- |
| 0-20 cm | CK | 4.98b (0.12) | 10.45b (0.10) | 0.22a (0.02) | 2.04b (0.30) | 64.33b (0.5) | 0.58b (0.03) | 0.89b (0.05) | 0.85c (0.02) | 32.20c (3.23) | 5.99b (0.19) | 30.81b (1.07) | 8.56c (0.08) | 7.19c (0.03) | 30.65c (0.20) |
|  | CF | 5.00b (0.09) | 12.83ab (0.18) | 0.26a (0.01) | 4.92ab (0.54) | 58.85b (3.05) | 0.82a (0.02) | 0.91b (0.02) | 1.13b (0.01) | 67.20a (1.62) | 7.03b (0.32) | 67.63a (4.68) | 9.13b (0.05) | 7.98b (0.02) | 35.15b (0.60) |
|  | OM | 7.07a (0.07) | 17.30a (0.08) | 0.28a (0.02) | 11.21a (1.20) | 179.07a (2.86) | 0.89a (0.02) | 1.13a (0.24) | 1.38a (0.02) | 53.67b (4.94) | 44.91a (0.58) | 29.56b (8.15) | 10.03a (0.11) | 8.72a (0.02) | 40.84a (0.64) |
| 20-40 cm | CK | 4.94b (0.06) | 9.01a (0.48) | 0.21b (0.03) | 3.27b (1.69) | 46.11b (3.01) | 0.66a (0.01) | 0.95b (0.02) | 0.62c (0.06) | 40.60b (3.23) | 5.00c (0.28) | 67.30a (3.96) | 7.94c (0.05) | 6.97c (0.04) |  |
|  | CF | 4.86b (0.06) | 8. 53a (0.18) | 0.19b (0.01) | 3.58b (0.80) | 79.51a (0.37) | 0.67a (0.01) | 0.65c (0.06) | 0.99b (0.03) | 66.73a (2.33) | 10.46b (0.46) | 46.43b (4.55) | 8.80b (0.08) | 7.39b (0.04) |  |
|  | OM | 7.06a (0.03) | 15.50a (0.50) | 0.34a (0.02) | 11.31a (0.84) | 82.35a (3.29) | 0.93a (0.03) | 1.20a (0.09) | 1.38a (0.01) | 37.33b (5.66) | 22.64a (0.55) | 21.05c (1.89) | 9.75a (0.08) | 7.93a (0.03) |  |
| 40-60 cm | CK | 4.96b (0.05) | 9.24a (2.92) | 0.22b (0.02) | 3.56a (3.26) | 46.01ab (4.53) | 0.61a (0.04) | 1.45a (0.01) | 0.66c (0.01) | 31.73c (2.60) | 5.05b (2.01) | 138.72a (3.04) | 7.56c (0.08) | 6.75c (0.04) |  |
|  | CF | 4.97b (0.24) | 7.25b (3.84) | 0.20b (0.02) | 4.35a (3.43) | 35.75b (3.31) | 0.61a (0.14) | 0.67b (0.01) | 0.85b (0.03) | 72.80a (2.84) | 22.85a (1.90) | 23.00b (2.18) | 8.44b (0.04) | 7.14b (0.07) |  |
|  | OM | 6.94a (0.39) | 9.06a (0.60) | 0.68a (0.04) | 3.93a (1.21) | 40.77a (1.76) | 0.68a (0.02) | 1.35a (0.01) | 1.31a (0.01) | 56.47b (3.06) | 21.70a (1.75) | 20.98b (1.16) | 8.92a (0.02) | 7.72a (0.03) |  |

Values in a row followed by the same letter in each soil depth are not significantly different at *P* < 0.05. Standard error is in parentheses. Bacterial or Fungal copy numbers were log10-transformed in abundance. SOC, soil organic carbon; DOC, dissolved organic carbon; POC, potassium permanganate-oxidizable carbon; MBC, microbial biomass carbon; TN, total soil nitrogen; AN, alkalyzable nitrogen; TP, Total phosphorus concentration; AP, available phosphorus; TK, total potassium concentration; AK, readily available potassium (the same below). CK, no fertilization; CF, chemical fertilization; OM, organic manure.

**Table S.5** Effects of fertilization and soil depth on soil microbial and nematode communities in ANOVA.

|  | Treatment | 2019 | | | | | | 2020 | | | | | |
| --- | --- | --- | --- | --- | --- | --- | --- | --- | --- | --- | --- | --- | --- |
|  |  | Bacteria | | Fungi | | Nematode | | Bacteria | | Fungi | | Nematode | |
|  |  | R^2^ | *P* | R^2^ | *P* | R^2^ | *P* | R^2^ | *P* | R^2^ | *P* | R^2^ | *P* |
| Soil depth | CK | 0.34 | 0.027 | 0.48 | 0.010 | 0.59 | 0.007 | 0.41 | 0.003 | 0.46 | 0.020 | 0.64 | 0.002 |
|  | CF | 0.37 | 0.028 | 0.58 | 0.005 | 0.64 | 0.016 | 0.38 | 0.014 | 0.37 | 0.023 | 0.61 | 0.003 |
|  | OM | 0.31 | 0.037 | 0.55 | 0.004 | 0.29 | 0.271 | 0.31 | 0.049 | 0.51 | 0.003 | 0.65 | 0.005 |
| Fertilization | 0-20 | 0.62 | 0.004 | 0.85 | 0.004 | 0.46 | 0.002 | 0.59 | 0.005 | 0.74 | 0.006 | 0.71 | 0.006 |
|  | 20-40 | 0.71 | 0.004 | 0.94 | 0.006 | 0.66 | 0.005 | 0.59 | 0.003 | 0.76 | 0.005 | 0.64 | 0.005 |
|  | 40-60 | 0.59 | 0.002 | 0.87 | 0.002 | 0.73 | 0.002 | 0.57 | 0.010 | 0.81 | 0.005 | 0.69 | 0.004 |

Values in a row in soil depth mean the effect of soil depth on soil microbial and nematode communities in each treatment. Values in a row in fertilization mean the effect of fertilization on soil microbial and nematode communities in each soil depth. CK, no fertilization; CF, chemical fertilization; OM, organic manure. 0-20, soil depth of 0-20 cm; 20-40, soil depth of 20-40 cm; 40-60, soil depth of 40-60 cm.

**Table S.6** Topological properties of co-occurring bacteria-fungi-nematode-SOC components networks obtained in treatments and their respective identically sized Erdös–Réyni random networks

| Network metrics | CK | CF | OM |
| --- | --- | --- | --- |
| Number of nodes | 35 | 33 | 37 |
| Number of edges | 158 | 124 | 215 |
| Number of positive correlations | 88 | 63 | 119 |
| Number of negative correlations | 70 | 61 | 96 |
| Percentage of the positive link (P%) of bacteria-fungi | 46.20 | 45.97 | 52.56 |
| P% of bacteria-nematode | 12.03 | 18.55 | 16.74 |
| P% of fungi-nematode | 4.43 | 4.03 | 3.26 |
| P% of bacteria-SOC components | 6.33 | 3.23 | 0.93 |
| P% of fungi-SOC components | 1.27 | 0.00 | 0.93 |
| P% of nematode-SOC components | 1.27 | 0.81 | 0.00 |
| P% of plant-parasites | 2.53 | 9.68 | 3.26 |
| Average connectivity (*avgK*) | 9.03 | 7.52 | 11.62 |
| Average clustering coefficient (*avgCC*) | 0.690 | 0.553 | 0.794 |
| Average path length (APL) | 2.43 | 2.09 | 2.27 |
| Network diameter | 8 | 4 | 7 |
| Graph density | 0.266 | 0.235 | 0.323 |
| Modularity (M) | 0.134 | 0.285 | 0.106 |
|  |  |  |  |
| *Random networks* |  |  |  |
| APL±s.d. | 1.80±0.02 | 1.89±0.01 | 1.69±0.01 |
| *avgCC*±s.d. | 0.25±0.02 | 0.22±0.01 | 0.32±0.01 |
| M±s.d. | 0.18±0.02 | 0.21±0.02 | 0.14±0.01 |

CK, no fertilization; CF, chemical fertilization; OM, organic manure. Percentage of the positive links to total links for each group is in parentheses.
